# Letter to the editor: indacaterol/glycopyrronium/mometasone furoate compared with salmeterol/fluticasone propionate in patients with asthma: a randomized controlled cross-over study

**DOI:** 10.1186/s12931-020-01349-5

**Published:** 2020-04-15

**Authors:** Henrik Watz, Jens M. Hohlfeld, Dave Singh, Jutta Beier, Zuzana Diamant, Jinming Liu, Shucheng Hua, Khalid Abd-Elaziz, Pascale Pinot, Ieuan Jones, Hanns-Christian Tillmann

**Affiliations:** 1 Pulmonary Research Institute at LungenClinic Grosshandorf, Airway Research Center North (ARCN), German Center for Lung Research (DZL), Woehrendamm 80, 22927 Grosshansdorf, Germany; 2 Fraunhofer Institute of Toxicology and Experimental Medicine and Respiratory Medicine of Hannover Medical School, Biomedical Research in End-Stage and Obstructive Lung Disease (BREATH), German Center for Lung Research (DZL), Hannover, Germany; 3 Medicines Evaluation Unit, University of Manchester, Manchester University NHS Foundation Trust, Manchester, UK; 4 Insaf Respiratory Research Institute, Biebricher Allee 34, 65187 Wiesbaden, Germany; 5 Department of Respiratory Medicine & Allergology, Institute for Clinical Science, Skane University Hospital, Lund University, 221 85 Lund, Sweden; 6 QPS-Netherlands, Groningen, The Netherlands; 7 Shanghai Pulmonary Hospital, Shanghai, China; 8 The First Hospital of Jilin University, Changchun City, Jilin Province China; 9 Novartis Institutes for Biomedical Research, Basel, Switzerland; 10 Novartis Pharma AG, Basel, Switzerland

**Keywords:** Indacaterol, Glycopyrronium, Mometasone Furoate, Asthma

## Abstract

**Abstract:**

Indacaterol (IND; 150 μg), glycopyrronium (GLY; 50 μg) and mometasone furoate (MF; 160 μg [high-dose ICS] and 80 μg [medium-dose ICS]) have been formulated as a once-daily (o.d.) fixed-dose combination treatment delivered via the Breezhaler® device for the treatment of patients with asthma. In this randomized (*n* = 116), double-blind, double-dummy, active comparator-controlled, three-period cross-over study we evaluated the benefit of o.d. IND/GLY/MF versus twice daily (b.i.d.) salmeterol/fluticasone propionate combination (SFC; 50/500 μg; high-dose ICS) treatment (NCT03063086). Overall, 107 patients completed the study. The study met its primary objective by demonstrating superiority of o.d. IND/GLY/MF at medium and high-dose ICS over b.i.d. SFC (high-dose ICS) in peak FEV_1_ after 21 days of treatment (+ 172 mL with high-dose and + 159 mL with medium-dose IND/GLY/MF versus SFC, *p* < 0.0001 for each comparison). We also observed that a higher percentage of patients did not need rescue medicine with IND/GLY/MF (high-dose ICS, 58%; medium-dose ICS, 52%) compared with SFC (45%) during the last week of each treatment period. Study treatments were well-tolerated with no relevant differences in tolerability between both IND/GLY/MF doses and SFC. In conclusion, both doses of IND/GLY/MF provided superior lung function benefits compared with twice-daily, standard-of-care SFC at the highest approved dose.

**Trial registration:**

ClinicalTrials.gov, (Identifier: NCT03063086),

EudraCT start date: May 11, 2017; First patient first visit / study initiation date: May 31, 2017.

**To the Editor:**

The combination of an inhaled corticosteroid (ICS) plus a long-acting β2-agonist (LABA) is considered standard-of-care therapy for patients with moderate-to-severe asthma (GINA step 3/4/5) [1]. However, some patients remain inadequately controlled despite using LABA/ICS combination treatments [2, 3]. Adding a long-acting muscarinic antagonist (LAMA) on top of LABA/ICS (medium- or high-dose ICS) can help to improve asthma outcomes in these patients [1, 4, 5].

The combination of the LABA indacaterol acetate (IND) and the LAMA glycopyrronium bromide (GLY) is presently available as once daily (o.d.) treatment for patients with chronic obstructive pulmonary disease (COPD). Recently, IND/GLY has been formulated in combination with the ICS mometasone furoate (MF) delivered via dry powder inhalation device (Breezhaler®) for the treatment of asthma.

We conducted a phase II multi-center study to investigate lung function parameters and rescue medication use with IND/GLY/MF compared with salmeterol/fluticasone propionate combination (SFC) in adults with asthma (NCT03063086). The primary study objective was to demonstrate superiority in peak bronchodilator effect of o.d. IND/GLY/MF (150/50/160 μg [high-dose ICS]; 150/50/80 μg [medium-dose ICS]) compared with twice-daily (b.i.d.) SFC (50/500 μg; high-dose ICS) after 21 days of treatment.

This confirmatory study had a randomized, double-blind, double-dummy, active-comparator-controlled, crossover design with three consecutive study periods of 21 treatment days each. Eligible patients were randomized to receive o.d. IND/GLY/MF (high-dose ICS; 160 μg MF), o.d. IND/GLY/MF (medium-dose ICS; 80 μg MF) and b.i.d. SFC (high-dose ICS) in one of six treatment sequences. The study was conducted in accordance with the Declaration of Helsinki and was approved by the Independent Ethics Committees of participating sites in Europe and China. Written informed consent was obtained from each patient before conducting any study specific procedures. Some results from this study have been previously reported in abstracts [6, 7].

Male and female patients with a documented physician diagnosis of asthma for a period of ≥12 months and who were previously treated with LABA/ICS combinations for ≥3 months and at a stable medium- or high-dose ICS for ≥1 month prior to screening were eligible to enrol. All patients had a pre-bronchodilator forced expiratory volume in 1 s (FEV_1_) < 80% of the predicted normal value (after withholding bronchodilators) and an FEV_1_ increase ≥12% and ≥ 200 mL after administration of 400 μg salbutamol/360 μg albuterol (or equivalent dose) at screening. Key exclusion criteria included current smokers or patients who had smoked tobacco products within 6 months prior to Visit 1 or who had a smoke history of greater than 10 pack years; patients who had an asthma exacerbation requiring systemic steroids, hospitalisation, or emergency room visit within 6 weeks prior to the study; and patients with a history of chronic lung diseases other than asthma.

From screening to the end of the study, patients were asked to record study medication intake, peak expiratory flow (PEF; a.m. and p.m.) and rescue medication use (short-acting β2-agonist [SABA], MDI; 100 μg salbutamol/90 μg albuterol) in an electronic diary (data from last week of each treatment period was pre-specified to be used for evaluation of PEF and rescue medication use). Spirometry measurements followed the American Thoracic Society (ATS)/European Respiratory Society (ERS) guidelines [8].

The sample size of this study was 116 randomized patients (52.6% male; mean age: 49.5 years [range: 18–76 years]), of which 107 patients completed the study. Adverse events (AEs) were the most common reason for discontinuation (3.4%). Other reasons included non-compliance with study treatment (1.7%), and patient and physician decision (both 0.9%). At baseline, mean pre-bronchodilator FEV_1_ was 2.2 L (range: 0.8–4.5 L), mean predicted FEV_1_ pre-bronchodilator was 62.2% (range: 25–82%pred.), mean SABA reversibility was 23.9% (range: 12–86%), and 90.5% of patients used LABA/ICS as prior medication (8.6% LABA/LAMA/ICS; 0.9% ICS only). Of 116 randomized patients, 86 were on medium-dose ICS at screening, 19 were on high-dose ICS, and 11 were on low-dose ICS; this gives an indication of the asthma severity of the study population.

The primary objective was met showing superiority of IND/GLY/MF at both ICS doses over SFC in peak FEV_1_ after 21 days of treatment (Fig. 1: 172 mL [95% CI: 137, 208; high-dose ICS]; 159 mL [95% CI: 123, 195; medium-dose ICS]; *p* < 0.0001). Similarly, on day 21 IND/GLY/MF (high- and medium-dose ICS) showed a superior treatment effect on mean FEV_1_ at all time points compared with SFC (Fig. 2). Both IND/GLY/MF doses also improved standardized FEV_1_AUC_5min-1h_ and FEV_1_AUC_5min-23h45min_ versus SFC (*p* < 0.0001; Fig. 1) and showed a superior treatment effect compared with SFC on mean PEF measurements (least square mean difference of 29 L/min [95% CI: 22, 35; *P* < 0.0001] with high-dose ICS and 24 L/min [95% CI: 18, 31; *P* < 0.0001] with medium-dose ICS versus SFC).

**Fig. 1:**
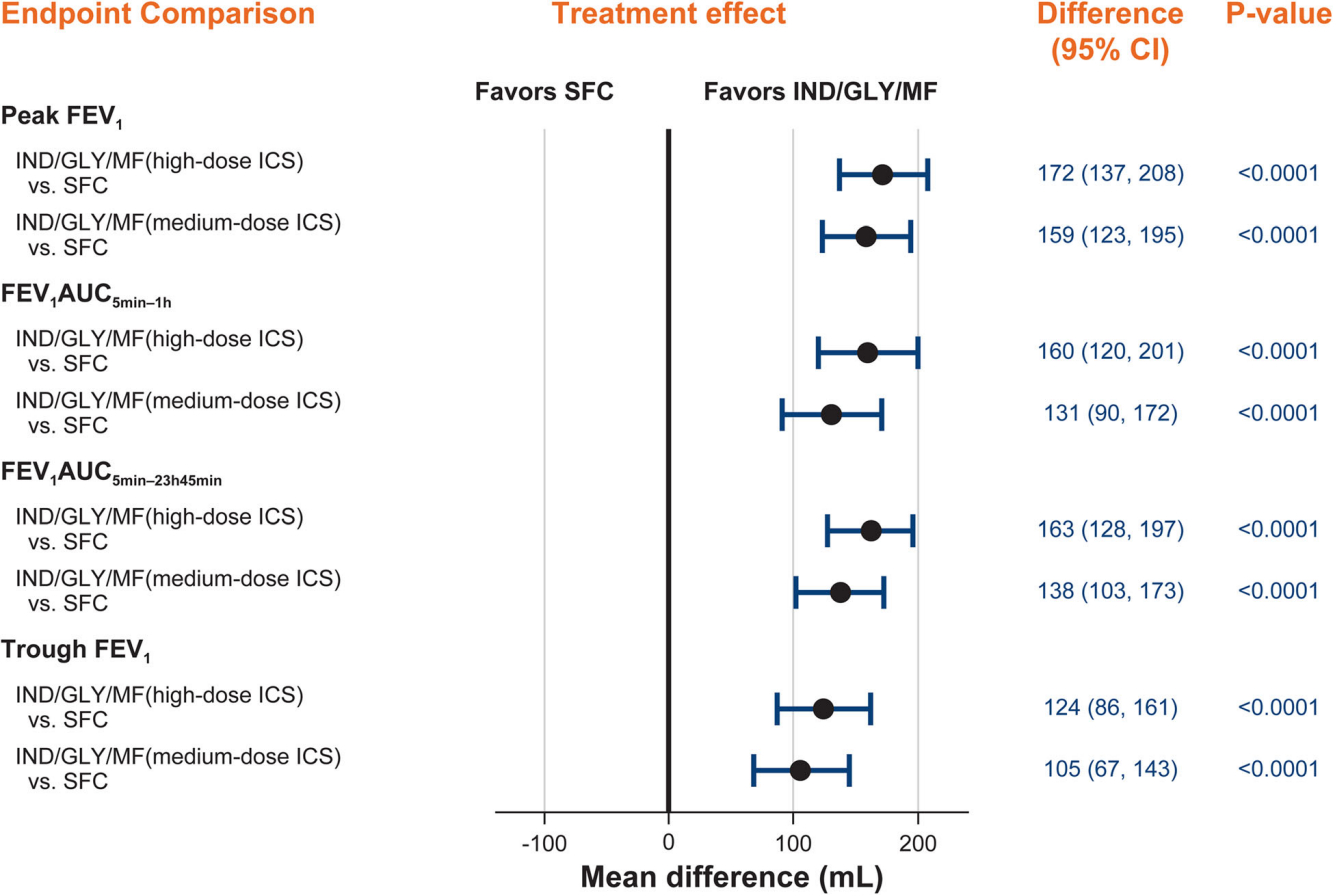
Effect of IND/GLY/MF (high- and medium-dose ICS) on peak FEV_1_ (L), standardized FEV_1_AUC_5min–1h_, FEV_1_AUC_5min–23h45min_, and trough FEV_1_ versus SFC (high-dose ICS) after 21 days of treatment. Peak FEV_1_ is defined as the highest bronchodilator effect on FEV_1_ during a period of 5 min to 4 h after the last evening dose of each treatment period. Standardized FEV_1_AUC is calculated as the area under the FEV_1_ curve over a specified time interval divided by the length of the time interval. Trough FEV_1_ is defined as the mean of FEV_1_ at 23 h 15 min and 23 h 45 min post-dose. CI: confidence interval; FEV_1_: forced expiratory volume in 1 s; GLY: glycopyrronium; ICS, inhaled corticosteroid; IND: indacaterol; MF: mometasone furoate; SFC, salmeterol/fluticasone combination

**Fig. 2:**
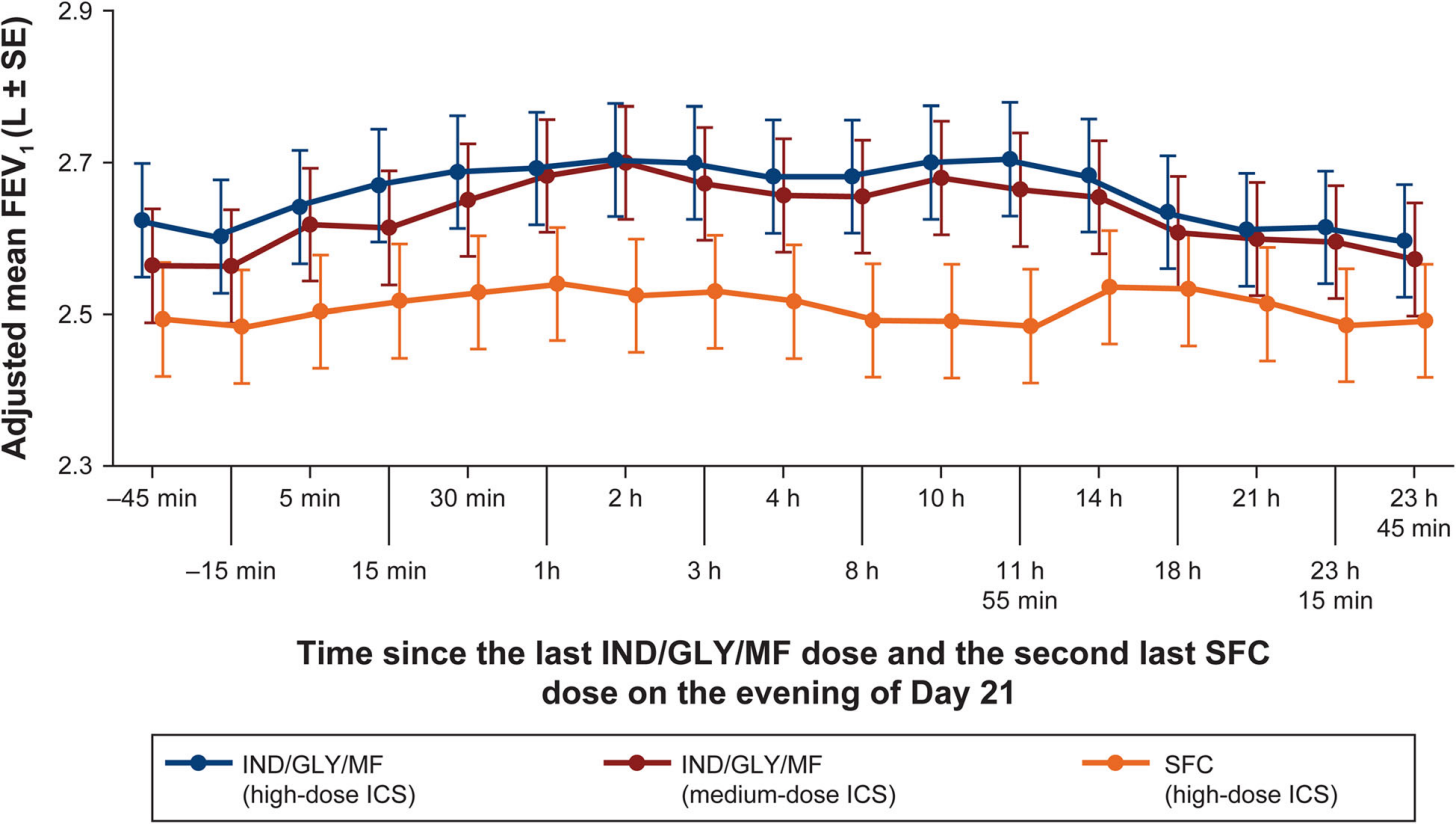
Adjusted mean FEV_1_ (L) by time point and treatment: after 21 days of treatment. *p* < 0.001 for comparisons between IND/GLY/MF (high and medium-dose ICS) and SFC (high-dose ICS) treatments at each time point after − 45 min (*p* < 0.001 for IND/GLY/MF [high-dose ICS] and *p* < 0.05 for IND/GLY/MF [medium-dose ICS] versus SFC at − 45 min). FEV_1_: forced expiratory volume in 1 s; GLY: glycopyrronium; IND: indacaterol; MF: mometasone furoate; SE: standard error; SFC, salmeterol/fluticasone combination

Furthermore, we observed that a higher percentage of patients did not need rescue medicine with IND/GLY/MF (high-dose ICS, 58%; medium-dose ICS, 52%) compared with SFC (45%). The odds of being free from rescue medication with IND/GLY/MF (high-dose ICS) was 2.4 times higher than with SFC (95% CI: 1.7, 5.0; *p* = 0.018). Similarly, the odds ratio (95% CI) was 1.7 (0.8, 3.4; *p* = 0.153) for IND/GLY/MF (medium-dose ICS) versus SFC.

Overall, study treatments were well-tolerated with no clinically relevant differences in AE rates (IND/GLY/MF [high-dose ICS]: 33.0%; IND/GLY/MF [medium-dose ICS]: 28.7%; SFC: 37.8%). The most commonly reported AEs included headache (IND/GLY/MF [high-dose ICS], 8.9%; IND/GLY/MF [medium-dose ICS], 8.7%; SFC, 11.7%), nasopharyngitis (2.7, 6.1, and 3.6%, respectively), cough (4.5, 2.6, and 2.7%, respectively), and dysphonia (5.4, 0.9, and 5.4%, respectively). Four (3.4%) patients discontinued the study due to AEs (tachyarrhythmia, diarrhea, asthma exacerbation during IND/GLY/MF [medium-dose ICS] treatment, and asthma exacerbation during SFC treatment). There were no serious AEs or new safety findings in the study.

In a previous study, with a different design and patient population, the open combination of tiotropium (Respimat®) 5 μg as add-on to LABA/ICS (high-dose ICS) increased mean peak FEV_1_ by 110 mL (95% CI: 63, 158) at week 24 compared with LABA/ICS (high-dose ICS) plus placebo [9]. In the present study, we report a least square mean peak FEV_1_ treatment difference of 172 mL and 159 mL for IND/GLY/MF high- and medium-dose ICS, respectively, when compared with SFC. Cross-study comparisons have substantial limitations due to differences in study design, treatment duration, and patient populations, therefore the authors caution against over-interpretation.

Although the lack of monitoring of asthma symptoms can be perceived as a limitation of this study, the decreased use of rescue medication during treatment with IND/GLY/MF versus SFC provides a signal for improved asthma control while on IND/GLY/MF.

While several studies with LABA/LAMA/ICS combination therapy in asthma are presently ongoing, our data provide evidence that a fixed-dose, once-daily treatment with IND/GLY/MF at medium- and high-dose ICS improves outcomes in patients with moderate-to-severe asthma in comparison to twice-daily high-dose ICS SFC.

## Data Availability

Novartis is committed to sharing with qualified external researchers, access to patient-level data and supporting clinical documents from eligible studies. These requests are reviewed and approved by an independent review panel on the basis of scientific merit. All data provided is anonymized to respect the privacy of patients who have participated in the study in line with applicable laws and regulations. This study data availability is according to the criteria and process described on www.clinicalstudydatarequest.com.

## Abbreviations

AE: Adverse events
ATS: American Thoracic Society
b.i.d.: twice-daily
CI: Confidence interval
COPD: Chronic obstructive pulmonary disease
ERS: European Respiratory Society
FEV_1_: Forced expiratory volume in 1 s
GLY: Glycopyrronium bromide
ICS: Inhaled corticosteroid
IND: Indacaterol acetate
LABA: long-acting β_2_-agonist
LAMA: Long-acting muscarinic antagonist
MF: Mometasone furoate
o.d.: Once daily
PEF: Peak expiratory flow
SABA: Short-acting β_2_-agonist
SFC: Salmeterol/fluticasone propionate combination
